# Supplementary figures and images for: Tissue- and Population-Level Microbiome Analysis of the Wasp Spider Argiope bruennichi Identified a Novel Dominant Bacterial Symbiont
Source: Microorganisms. 2019 Dec 19;8(1):8. doi: 10.3390/microorganisms8010008 (PMC7023434; doi:10.3390/microorganisms8010008)

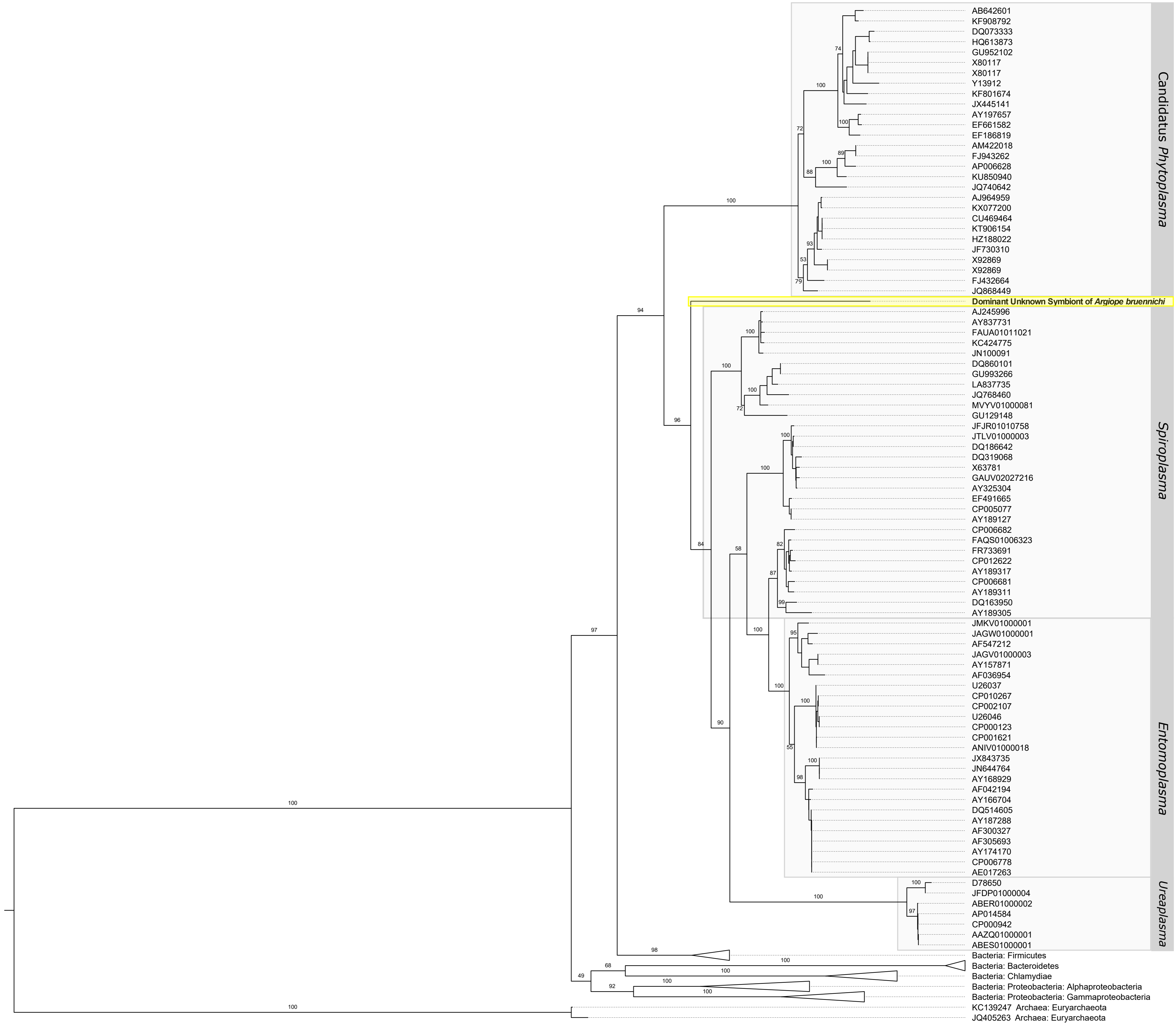

Supplement: Supplementary file 1 [file microorganisms-08-00008-s001.zip › Sheffer_RevisedSupplement/Supplementary Figure S1_GeneTree.pdf]
